# Comparative Efficacy of Non‐Transplant Interventions for Female Pattern Hair Loss: A Systematic Review and Network Meta‐Analysis

**DOI:** 10.1111/jocd.71037

**Published:** 2026-07-12

**Authors:** Caixia Hu, Xin Li, Wenqing Wang, Xiaomei Han, Lijing Lv, Molin Yang, Yang Gao, Yi Cheng

**Affiliations:** ^1^ Department of Dermatology The Fourth Hospital of Hebei Medical University Shijiazhuang China; ^2^ Interventional Department Hebei Children's Hospital Shijiazhuang China

**Keywords:** combination therapy, comparative efficacy, female pattern hair loss, network meta‐analysis, non‐transplant treatment

## Abstract

**Objective:**

Female pattern hair loss (FPHL) imposes significant psychosocial burdens, yet optimal non‐transplant strategies remain undefined due to limited comparative evidence. This study aimed to evaluate the comparative efficacy of active non‐transplant interventions for FPHL through a systematic review and network meta‐analysis (NMA).

**Methods:**

Randomized controlled trials (RCTs) assessing FPHL treatments were identified through systematic searches of PubMed, Embase, Cochrane Library, CNKI, WanFang, VIP, and SinoMed (inception to 1 January 2025). Risk of bias was assessed using the Cochrane tool. NMA was performed in R, with interventions ranked by SUCRA. Evidence certainty was evaluated using GRADE for NMAs. The certainty of the evidence was assessed using the GRADE approach.

**Results:**

Fourteen RCTs involving 919 patients were included. For hair density, microneedling plus minoxidil (MN + MX) ranked first (SUCRA = 0.93) and was potentially more effective than MX (MD = 21.13; 95% CrI, 15.38 to 27.30) and spironolactone plus minoxidil (SPT + MX) (MD = 14.05; 95% CrI, 5.44 to 22.85). For hair diameter, poly‐L‐lactic acid threads + minoxidil (PLLA+MX) was more effective than MX (MD = 43.63; 95% CrI, 15.35 to 71.80) and ranked first (SUCRA = 0.97). MN + MX also ranked first in patient satisfaction (SUCRA = 0.98). However, the certainty of all evidence was low to very low due to risk of bias, imprecision, and sparse networks. Importantly, as no placebo or untreated controls were included, findings reflect only relative rankings among active interventions. Subgroup analysis and safety analysis were not performed due to inconsistent reporting and the limited number of studies.

**Conclusion:**

MN + MX and PLLA+MX showed the highest relative rankings for hair density and diameter, respectively. Nonetheless, given the substantial uncertainty, these findings are exploratory and necessitate validation in larger, high‐quality placebo‐controlled trials.

## 1 Introduction

Female pattern hair loss (FPHL), also known as female androgenetic alopecia (FAGA) [1, 2], is a common condition affecting millions of women worldwide. It typically presents as progressive thinning and reduced hair density primarily over the vertex scalp, often leading to psychological distress, including anxiety, depression, and impaired quality of life—particularly in cases with widespread involvement [2, 3]. Unlike male pattern hair loss, the association between FPHL and hormonal imbalances is often less pronounced, complicating diagnosis and treatment [4]. Prevalence increases with age and exhibits significant ethnic variation, affecting approximately 19%–25% of Caucasian women compared to 6%–12% of Asian and even fewer African populations [5, 6].

Topical minoxidil is the only approved pharmacological therapy, yet many patients experience suboptimal results. As a result, non‐transplant adjunctive treatments—such as low‐level laser therapy (LLLT), microneedling, platelet‐rich plasma (PRP), and oral antiandrogens like spironolactone—are increasingly used. Newer approaches, including poly‐L‐lactic acid (PLLA) thread implantation, aim to stimulate hair growth through dermal remodeling [7, 8]. Clinical practice increasingly favors early combination therapies to improve outcomes, with growing interest in personalized treatment strategies [9].

Despite increasing clinical use, the relative efficacy of these interventions remains uncertain due to a scarcity of head‐to‐head randomized controlled trials. Network meta‐analysis (NMA) integrates direct and indirect evidence to simultaneously compare multiple interventions within a unified framework, enabling a comprehensive assessment of relative efficacy even in the absence of direct comparisons. However, prior network meta‐analyses on androgenetic alopecia have largely focused on male‐dominant or mixed‐sex populations, which limits their applicability to women because of sex‐specific differences in pathophysiology, hormonal profiles, and treatment response [10, 11, 12, 13].

Hence, our analysis included only female participants to provide evidence‐based insights specifically for FPHL management. To inform individualized treatment strategies, we explored three complementary outcomes that reflected distinct dimensions of therapeutic benefits: hair density, hair diameter, and patient‐reported satisfaction. While objective structural metrics like hair density and diameter are often subject to diverse measurement techniques, patient satisfaction captures subjective, appearance‐related concerns that are central to clinical success. Our aim was to generate an exploratory evidence‐based ranking of available treatments among active clinical options to support dermatologists in making informed, individualized decisions for women with FPHL.

## 2 Methods

This study was reported in accordance with the Preferred Reporting Items for Systematic Reviews and Meta‐Analyses (PRISMA) statement and its extension for network meta‐analyses (PRISMA‐NMA) [14]. The analysis adhered to the pre‐registered protocol (PROSPERO number: CRD420250630804), in which subgroup analyses were not planned due to insufficient numbers of eligible randomized controlled trials.

### 2.1 Literature Search

PubMed, Embase, Cochrane Library, China National Knowledge Infrastructure (CNKI), Wanfang, VIP and SinoMed were systematically searched from inception to Jan 1, 2025. The search terms were androgenetic alopecia, hair loss, pattern baldness, baldness, female pattern, female pattern alopecia, females, female, randomized controlled trial, and randomized. The search strategy is detailed in Table S1.

### 2.2 Eligibility Criteria

#### 2.2.1 Inclusion Criteria

1. Participants: Adult females with clinician‐diagnosed FPHL
2. Comparators: Head‐to‐head comparisons between two or more active non‐transplant interventions within randomized controlled trials (RCTs). To ensure a focused comparison of clinical therapeutic options, placebo or no‐treatment arms were excluded from the final network analysis. However, placebo or no‐treatment arms were excluded. Consequently, all effect estimates reflected relative rankings among active treatments only.
3. Interventions: Active non‐transplant therapies for FPHL, including both monotherapies (e.g., topical or oral medications, device‐based therapies) and combination therapies (e.g., pharmacological plus physical modalities).
4. Outcome: The primary outcome was the change in hair density (hairs/cm^2^), while secondary outcomes included changes in hair diameter (μm) and patient‐reported satisfaction (assessed via Likert‐type scales ranging from “very dissatisfied” to “very satisfied”). Safety data were extracted narratively; however, a quantitative synthesis was not performed due to substantial heterogeneity in safety reporting formats across trials.
5. Language: Only studies published in English or Chinese were included to ensure accurate data extraction and interpretation.

#### 2.2.2 Exclusion Criteria

1. Non‐randomized studies, transplant interventions, preclinical research, review articles, duplicates, conference abstracts.
2. RCTs that compared only a single active intervention against placebo or no treatment.
3. Studies with insufficient or non‐extractable data on efficacy or safety data.

### 2.3 Study Selection and Data Extraction

Study selection was conducted independently by two reviewers. Duplicate records were removed using EndNote X9, followed by screening of titles and abstracts. Potentially eligible studies proceeded to full‐text assessment against the predefined inclusion criteria. Disagreements were resolved through discussion and consensus, with arbitration by a third, senior reviewer when necessary. Substantial agreement was observed between reviewers during study selection, and inter‐rater reliability was not formally calculated due to the small number of included studies.

Data were extracted using a standardized form and included the following: first author, publication year, study design, intervention details, sample size, participant age, baseline hair loss severity, and primary outcome measures. All studies used trichoscopic or dermoscopic imaging with either automated or manual analysis to assess hair density and diameter. Despite this common approach, heterogeneity in imaging protocols (magnification, polarization), scalp sampling sites, terminal hair definitions, and analytical methods may limit comparability of effect estimates. This variability was acknowledged in the GRADE assessment as a source of imprecision and is noted as a limitation.

### 2.4 Quality Assessment

Risk of bias was assessed using the Cochrane Risk of Bias tool for randomized trials (RoB 2) [15]. Two reviewers independently evaluated five domains: randomization process, deviations from intended interventions, missing outcome data, measurement of the outcome, and selection of the reported result. Discrepancies were resolved by a third reviewer.

### 2.5 Data Analysis

Network meta‐analysis was conducted using a Bayesian framework in R with the ‘gemtc’ (v1.0‐2) and ‘R2jags’ packages. Multi‐arm trials were treated as single units to preserve within‐study correlations through shared baseline parameters. A random‐effects model was pre‐specified for all analyses due to expected clinical heterogeneity (e.g., variations in formulations, follow‐up duration, and baseline severity). Model convergence was confirmed via Potential Scale Reduction Factor (*PSRF* < 1.05) and trace/density plots. The transitivity assumption was rigorously assessed by comparing the distribution of potential effect modifiers (e.g., age, baseline severity, and treatment duration) across treatment comparisons. Consistency was evaluated using global design‐by‐treatment interaction and local node‐splitting models. Furthermore, potential procedural bias arising from diverse measurement techniques (manual vs. automated) was formally addressed using sensitivity analysis, restricting the evidence base to high‐quality studies utilizing automated trichoscopy. Treatments were ranked based on surface under the cumulative ranking curve (SUCRA) values (0%–100%). Higher SUCRA values indicated a greater probability of high relative efficacy [16]. However, given the sparse evidence network and wide 95% CrIs in certain nodes, SUCRA rankings were consistently framed as highly uncertain exploratory findings of relative efficacy rather than definitive evidence of absolute clinical superiority. Pre‐specified subgroup analyses were not performed due to limited data.

## 3 Results

### 3.1 Literature Search Results

**FIGURE 1:**
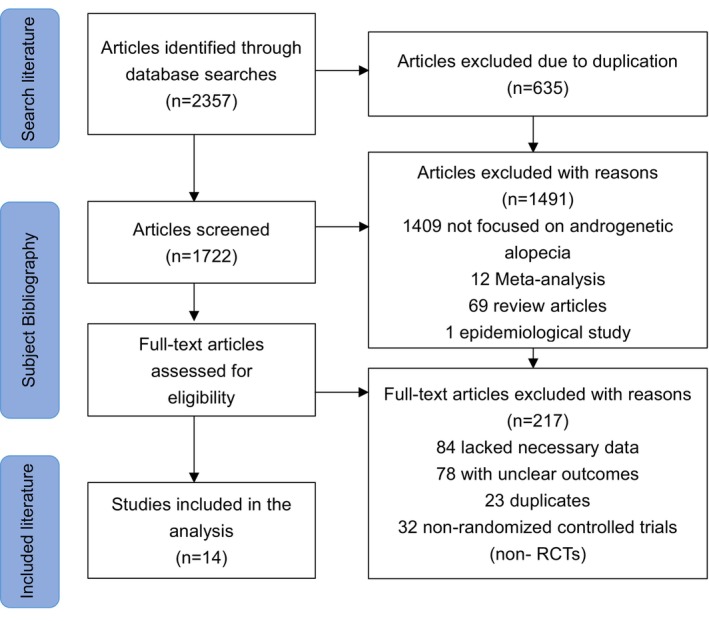
Literature search flowchart.

A comprehensive systematic review of the extant literature yielded a total of 2357 records: 581 from PubMed, 216 from Embase, 1314 from the Cochrane Library, 113 from CNKI, 19 from Wanfang, 11 from VIP, and 103 from SinoMed. After removing 635 duplicate records, 1722 unique citations were screened for eligibility. Of these, 1491 were excluded during title and abstract screening, leaving 231 articles for full‐text assessment. Following detailed evaluation, 217 studies were excluded for failing to meet the inclusion criteria, resulting in 14 RCTs [17, 18, 19, 20, 21, 22, 23, 24, 25, 26, 27, 28, 29, 30] eligible for inclusion in the analysis. The detailed screening process is shown in Figure 1.

### 3.2 General Characteristics

A total of 14 RCTs involving 919 patients with FPHL were included. The study by Bassiouny et al. [18] was analyzed as two separate trials due to independent assessments of frontal and parietal scalp regions. Participants' ages ranged from 20 to 75 years across studies. The disease severity was classified according to Ludwig (I–III), Savin (III–VI), and Sinclair (II–V) scales. The majority of participants exhibited Ludwig I–II, Sinclair II–III, or Savin III–IV grades, representing a predominantly mild‐to‐moderate severity. In terms of study design, five trials utilized three‐arm designs, while the remainder employed two‐arm designs. Crucially, all included studies utilized active comparators; no placebo or no‐treatment arms were evaluated within the evidence network. Topical minoxidil (MX) was the most common comparator, serving as the reference therapy in 13 studies. Follow‐up periods were largely consistent, with 92.8% (13/14) of studies evaluating outcomes at 24 weeks. This network meta‐analysis evaluates 15 active therapeutic strategies. Combination therapies included microneedling plus minoxidil (MN + MX), microneedling plus YU FA Scalp Nutrient Solution (MN + YF), spironolactone plus minoxidil (SPT + MX), low‐level light therapy (LLLT) combined with platelet‐rich plasma (PRP) or minoxidil, topical 1% cetirizine plus minoxidil (C + MX), polylactic acid thread therapy plus minoxidil (PLLA+MX), and topical 0.25% finasteride plus minoxidil (F + MX). Monotherapies evaluated were topical minoxidil solution (MX) or foam (MTF), oral minoxidil (OM) 1 mg or 0.25 mg once daily, LLLT, PRP, and topical cow placenta extract lotion (cow placenta). Detailed characteristics are summarized in Table 1, and the systematic assessment of potential effect modifiers (baseline severity, duration, and protocols) is provided in Table S2 to support the transitivity assumption.

**TABLE 1:** General characteristics of the included RCTs.

Study	Intervention	Dose	Sample size (*n*)	Alopecia grading (*n*)	Age (years)	Treatment time (weeks)	Outcomes
Blume 2011	5% minoxidil	50 mg	56	Savin III:15, IV:22, V:16, VI:3	23–68	24	①②
	2% minoxidil	1 mL	57	Savin III:20, IV:28, V:7, VI:2	25–75		
Suchonwanit 2018	0.25% finasteride +3% minoxidil	1 mL	15	Ludwig I:5, II:8, III:2	56.8 ± 6.6	24	①②
	3% minoxidil	1 mL	15	Ludwig I:4, II:8, III:3	59.8 ± 7.7		
Ramos 2020	Oral Minoxidil 1 mg tablets	1 mg	26	Sinclair I:15, II:10, III:1	40.6 ± 12.0	24	①②
	5% minoxidil	Once/day	26	Sinclair I:17, II:8, III:1	47.3 ± 11.8		
Yang liu 2020	Low dose laser therapy	Once/day	28	Ludwig I:7, II:15, III:6	20–48	24	①②③
	5% minoxidil	1 mL	27	Ludwig I:5, II:14, III:8	21–47		
	Low dose laser therapy+5% minoxidil	Once/day+1 mL	28	Ludwig I:6, II:14, III:8	20–49		
Liang 2022	5% minoxidil	1 mL	38	SinclairII, III	31.08 ± 6.87	24	①②
	5% minoxidil +Spironolactone	1 mL + 80–100 mg	37		31.62 ± 6.29		
	5% minoxidil +microneedle	1 mL twice/week	40		30.83 ± 6.28		
Xuelei liang 2022	5% minoxidil	1 mL	38	SinclairII, III	31.08 ± 6.87	24	①②③
	5% minoxidil +microneedle	1 mL twice/week	40		30.83 ± 6.28		
	Field generation fluid + microneedle	1 mL+ twice/week	38		31.66 ± 6.72		
Zhang 2022	2% minoxidil	1 mL	20	Sinclair II:7, III:10, IV:3	30.05 ± 5.46	24	①
	2% minoxidil +microneedles	1 mL + twice/week	20	Sinclair II:8, III:9, IV:3	31.68 ± 4.93		
Barat 2020	2% minoxidil	1 mL^2^	31	SinclairII~ V	41.29 ± 10.41	24	①
	Bovine placenta serum extract		43		44.39 ± 11.44		
Vahabi 2021	Oral Minoxidil 0.25 mg tablets	0.25 mg	26	SinclairII~ V	18–50	36	①②
	2% minoxidil	1 mL	25				
Nascimento 2022	Oral Minoxidil 0.25 mg tablets	0.25 mg	14	SinclairII~ V	41.70 ± 13.50	24	①
	Oral Minoxidil 1 mg tablets	1 mg	12		41.40 ± 11.00		
Yang liu 2020	Low dose laser therapy	Once/day	18	Ludwig I:5, II:10, III:3	30.86	24	①②
	Platelet‐rich plasma	Once/month	19	Ludwig I:5, II:11, III:3	30.62		
	Platelet‐rich plasma+ Low dose laser therapy	Once/day+ Once/month	19	Ludwig I:4, II:11, III:4	29.82		
Khattab 2019	Monofilament therapy +2% minoxidil	1 mL	27	Ludwig I:3, II:10, III:10	21–49	24	①③
	2% minoxidil	1 mL	27				
Bassiouny 2022 (forehead)	5% minoxidil+1% cetirizine	1 mL	26	Sinclair II:9, III:10, IV:7	38.61 ± 8.74	24	①
	5% minoxidil	1 mL	27	Sinclair II:16, III:7, IV:4	36.74 ± 9.84		
Bassiouny 2022 (vertex)	5% minoxidil+1% cetirizine	1 mL	26	Sinclair II:9, III:10, IV:7	38.61 ± 8.74	24	①
	5% minoxidil	1 mL	27	Sinclair II:16, III:7, IV:4	36.74 ± 9.84		
Esmat 2017	5% minoxidil	1 mL	23	Ludwig I:6, II:6, III:3	31.27 ± 5.57	24	①②
	Low dose laser therapy	Once/day	23	Ludwig I:2, II:10, III:3	35.67 ± 7.17		
	Low dose laser therapy+5% minoxidil	1 mL + once/day	23	Ludwig I:3, II:7, III:5	35.2 ± 8.86		

*Note:* Only outcome indicators included in this study are provided. ① Represents change in hair density; ② Represents change in hair diameter; and ③ Represents patient satisfaction with hair growth.

### 3.3 Quality Assessment

**FIGURE 2:**
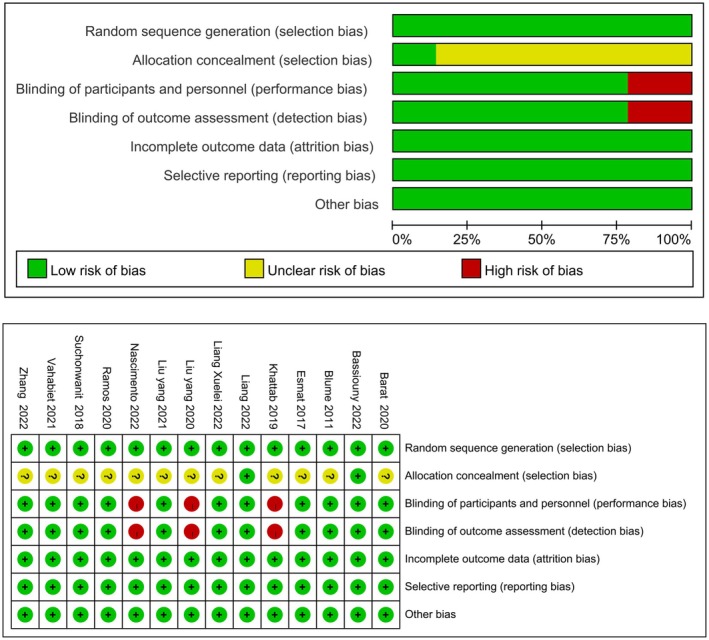
Risk of bias assessment results for the included randomized controlled trials.

The majority of studies demonstrated adequate random sequence generation; however, the allocation concealment process was rated as unclear in 12 of the 14 trials. The blinding of participants and personnel was robustly reported in 11 studies, while three were at high risk of bias due to open‐label designs or lack of investigator blinding. Furthermore, the blinding of outcome assessment was unclear or inadequate in three studies, potentially impacting the reliability of subjective outcomes such as patient‐reported satisfaction. While the majority of studies demonstrated a low risk of bias with regard to incomplete outcome data and selective reporting, the effect estimates should be carefully interpreted due to the unclear allocation concealment and inconsistent blinding. Comprehensive risk‐of‐bias assessments are illustrated in Figure 2.

### 3.4 Results of Network Meta‐Analysis

#### 3.4.1 Change in Hair Density(Hairs/cm^2^)

**FIGURE 3:**
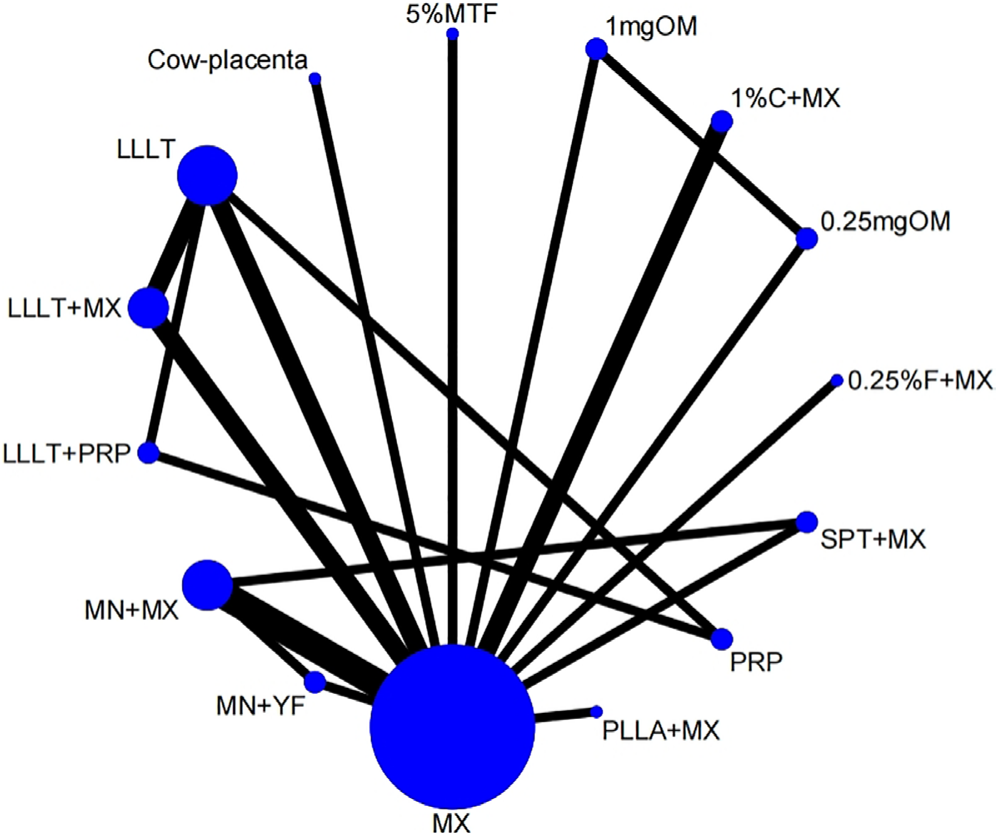
Network diagram for change in hair density. MX, topical minoxidil; MTF, topical minoxidil foam; OM, oral minoxidil once daily; LLLT, low‐level laser therapy; PRP, platelet‐rich plasma; MN, microneedling; SPT, spironolactone; C, topical 1% cetirizine; F, topical 0.25% finasteride solution; PLLA, polylactic acid thread therapy; YF, YU FA Scalp Nutrient Solution; cow placenta, topical cow placenta.

A network of 14 RCTs (five three‐arm and nine two‐arm) evaluating 15 active interventions was synthesized, encompassing 19 direct pairwise comparisons (Figure 3). All Bayesian models achieved satisfactory convergence (*PSRF* = 1.00; trace and density plots showed stable, well‐mixed chains), and no evidence of significant inconsistency was detected. Under the consistency model, MN + MX demonstrated a statistically significant increase in hair density compared to seven other interventions, including PLLA + MX (MD = 22.52; 95% CrI: 5.38 to 39.30), 1% C + MX (MD = 22.50; 95% CrI: 5.63 to 39.11), cow placenta (MD = 21.83; 95% CrI: 11.97 to 32.37), MX (MD = 21.13; 95% CrI: 15.38 to 27.30), LLLT (MD = 20.30; 95% CrI: 11.40 to 29.94), 5% MTF (MD = 17.71; 95% CrI: 5.90 to 30.04), and LLLT + PRP (MD = 14.87; 95% CrI: 2.93 to 27.66) (Table S3). Consistent with these relative rankings, MN + MX showed the highest relative probability of efficacy (SUCRA = 0.93), followed by PLLA + MX (SUCRA = 0.88).

However, these rankings must be interpreted with caution due to several critical factors. Firstly, due to the absence of placebo arms in clinical trials, it was impossible to draw definitive conclusions, without establishing absolute therapeutic efficacy. Secondly, while SUCRA value suggests a clear trend, given the overlapping credible intervals (CrIs) in the league table (e.g., between MN + MX, PLLA+MX, and SPT + MX), the comparative superiority of many top interventions remained statistically indistinguishable. Furthermore, due to the wide CrIs observed in certain nodes and the heterogeneity in baseline severity and measurement protocols across studies, the evidence in this network was highly uncertain. The complete hierarchy of interventions was: MN + MX>PLLA + MX>LLLT + MX>SPT + MX > 1 mg OM>LLLT + PRP > 5% MTF > 0.25% F+ MX>LLLT > 0.25 mg OM>MX > 1% C + MX>cow placenta>PRP > MN + YF (Figure S1, Table S4).

#### 3.4.2 Change in Hair Diameter (μm)

**FIGURE 4:**
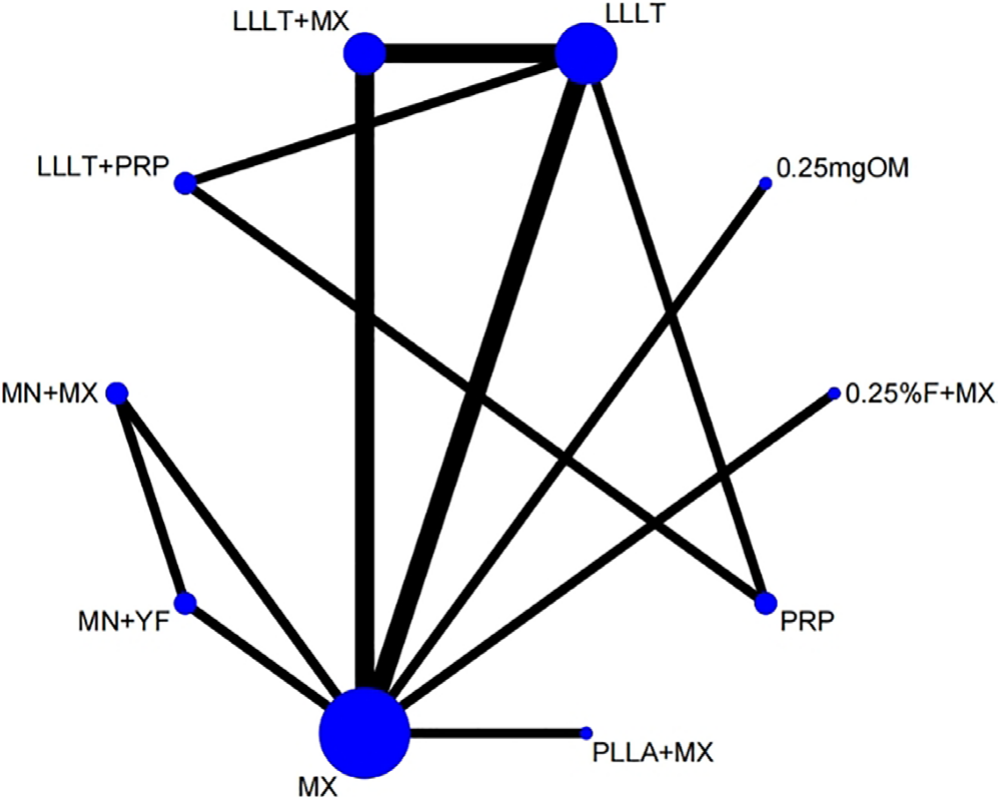
Network diagram for change in hair diameter. Abbreviations as in Figure 3.

A network of seven RCTs (four three‐arm and three two‐arm) evaluating 10 active interventions formed a network of 12 direct comparisons (Figure 4). All models achieved satisfactory convergence (*PSRF* = 1.00; trace and density plots showed stable, well‐mixed chains), and no evidence of significant inconsistency was detected. Under the consistency model, PLLA+MX demonstrated a statistically significant increase in hair diameter compared to four interventions: MN + YF (MD = 45.64; 95% CrI: 5.34 to 85.08), MX (MD = 43.63; 95% CrI: 15.35 to 71.80), 0.25 mg OM (MD = 42.64; 95% CrI: 2.53 to 82.38), and MN + MX (MD = 42.62; 95% CrI: 2.42 to 82.25) (Table S5).

According to SUCRA rankings, PLLA + MX possessed the highest probability of efficacy for improving hair diameter (SUCRA = 0.97), followed by LLLT + MX (SUCRA = 0.67). However, these hierarchical rankings must be interpreted as exploratory and with extreme caution. The evidence base for this outcome was markedly sparse (only seven trials), leading to highly imprecise effect estimates with exceptionally wide 95% CrIs (e.g., the lower bound for PLLA+MX vs. MN + MX is as low as 2.42). This lack of certainty suggested that the comparative advantage of many interventions remains statistically indistinguishable, and these rankings should not be viewed as robust clinical hierarchies. The complete hierarchy was: PLLA + MX>LLLT + MX>LLLT + PRP>PRP > LLLT > 0.25% F + MX>MN + MX > 0.25 mg OM>MX > MN + YF (Figure S2, Table S4).

#### 3.4.3 Patient Satisfaction

**FIGURE 5:**
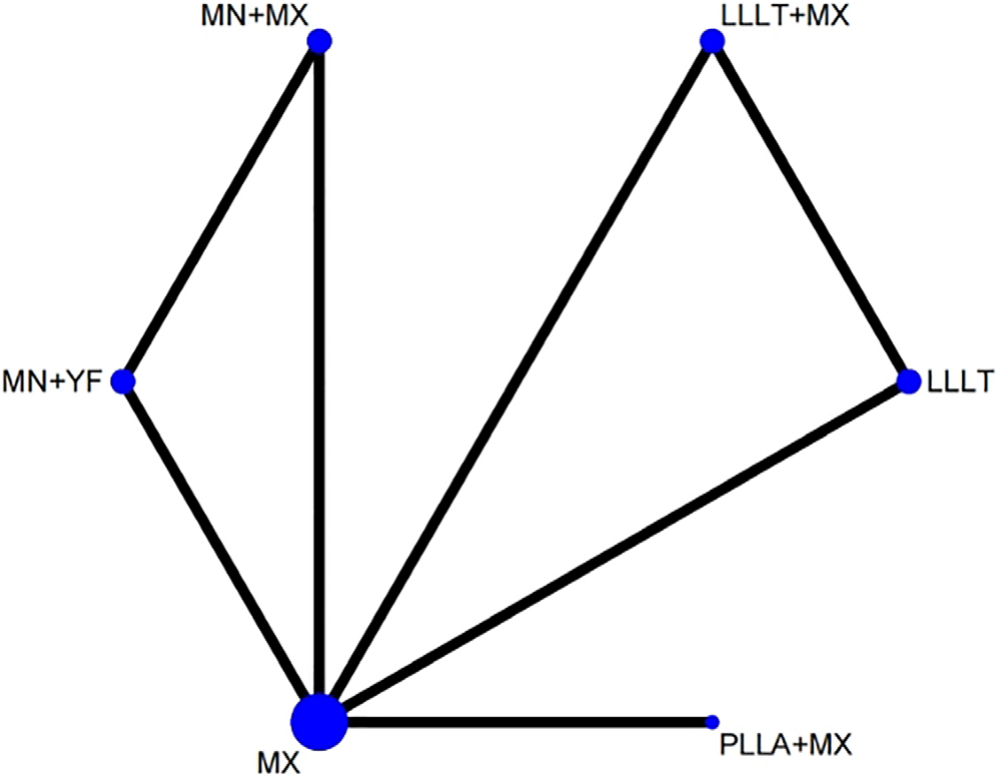
Network diagram for patient satisfaction. Abbreviation as in Figure 3.

A network for patient satisfaction was synthesized from three active‐controlled RCTs (two three‐arm and one two‐arm) evaluating six interventions, encompassing seven direct comparisons (Figure 5). All models achieved satisfactory convergence, and no significant inconsistency was detected. Under the consistency model, MN + MX (Log OR = 2.96; 95% CrI: 0.06 to 5.86) and PLLA+MX (Log OR = 2.45; 95% CrI: 0.83 to 4.08) demonstrated statistically significant patient‐reported satisfaction compared to MX (Table S6). Furthermore, PLLA+MX showed a higher Log OR than LLLT (Log OR = 2.45; 95% CrI: 0.12 to 4.79).

According to SUCRA values, MN + MX (0.98) and PLLA+MX (0.58) ranked high (Table S4). However, in strict accordance with the transitivity and interpretability concerns raised, these rankings were considered highly uncertain. The evidence base for this outcome was markedly constrained (*n* = 3 studies). Given no placebo or no‐treatment arms, these findings only indicated a relative ranking, without establishing absolute efficacy. Furthermore, the reliance on limited, non‐validated patient satisfaction scales across trials introduces substantial methodological noise. Furthermore, due to the wide and overlapping credible intervals, we cannot draw any definitive conclusions regarding comparative clinical superiority. Consequently, these SUCRA rankings should be regarded as exploratory qualitative trends only, and are inadequate for guiding clinical decision‐making. The complete ranking of interventions was as follows: MN + MX > PLLA+MX > LLLT+MX > MN + YF > MX > LLLT (Figure S3, Table S4).

### 3.5 Assessment of Publication Bias

**FIGURE 6:**
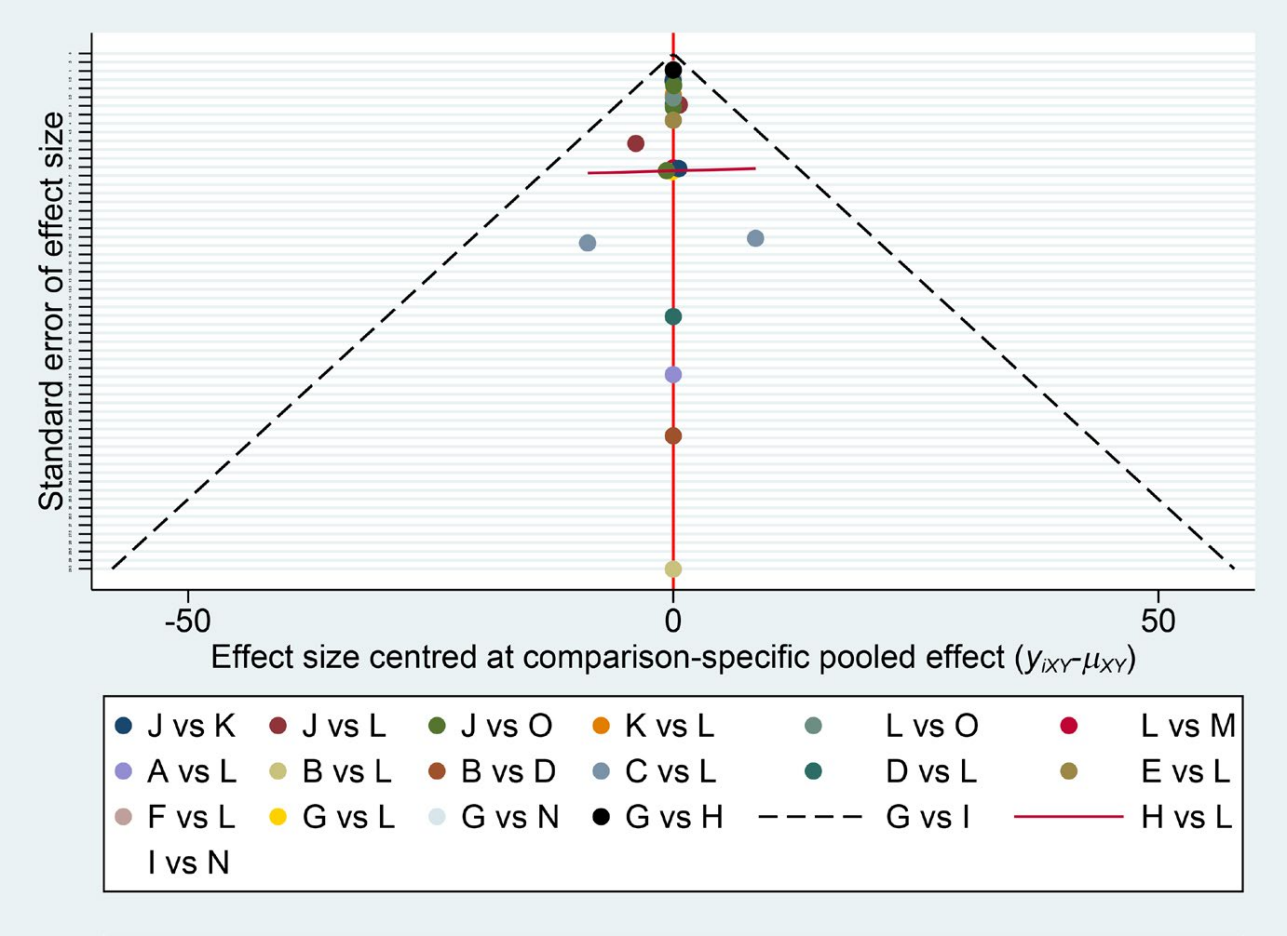
Funnel plot for change in hair density. Interventions are labeled as follows: A, MX; B, 0.25 mg OM; C, 1 mg OM; D, MTF; E, C + MX; F, cow placenta; G, LLLT; H, LLLT + MX; I, LLLT + PRP; J, MN + MX; K, MN + YF; L, F + MX; M, PLLA + MX; N, PRP; O, SPT + MX. Full intervention names and abbreviations are provided in Figure 3.

The funnel plot for the hair density outcome (Figure 6) demonstrated a generally symmetrical distribution of studies, primarily clustered in the middle to upper regions. However, given the relatively small number of included studies (*n* = 14), the statistical power to detect publication bias remains inherently limited. Consequently, this visual symmetry should be interpreted with caution as a preliminary observation rather than definitive evidence of a low risk of publication bias. One study was identified as a potential outlier in the lower portion of the funnel, which may suggest a small‐study effect, inherent methodological differences, or stochastic variation. For the outcomes of hair diameter and patient satisfaction, a formal assessment of publication bias via funnel plots was not performed due to the insufficient number of trials (*n* < 10) in those networks, which would render the plots uninterpretable.

### 3.6 Robustness and Certainty of Evidence

#### 3.6.1 Sensitivity Analysis and Outcome Stability

To evaluate the robustness of our primary findings, we performed sensitivity analyses by focusing on studies utilizing automated trichoscopy and those with low‐to‐moderate RoB. Due to the resulting sparse networks, pairwise meta‐analyses were used as a robust alternative to validate the consistency of the rankings (Tables S7–S11).

Regarding hair density, the relative advantage of MN + MX over MX was consistently sustained across both high‐quality subsets. Conversely, the primary ranking of PLLA+MX for hair diameter could not be confirmed, as the sole supporting study by Khattab et al. [21] was excluded from the sensitivity analysis due to a high risk of bias. Furthermore, while LLLT exhibited a notable advantage in hair diameter, LLLT+MX did not demonstrate a comparable effect in the restricted subsets. These sensitivity analysis results confirmed the stability of the MN + MX findings while highlighting the fragility of the evidence supporting PLLA+MX.

#### 3.6.2 GRADE Certainty of Evidence and Clinical Implications

In accordance with the GRADE framework, the certainty of the evidence for all key comparisons was evaluated as low to very low, primarily due to limitations in blinding (risk of bias), small sample sizes (imprecision), and the indirectness inherent in the active‐controlled network (Table S12). The sensitivity analyses also indicated a considerable degree of residual uncertainty in the findings. Consequently, while the observed benefits of MN + MX across multiple outcomes were promising, they should be interpreted as preliminary and hypothesis‐generating rather than definitive findings. Given the current evidence base, we cannot endorse MN + MX as a first‐line combination strategy in routine clinical practice without further high‐quality justification. Large‐scale, multi‐center RCTs employing standardized measurement protocols and placebo arms are essential to substantiate these observations.

### 3.7 Safety and Adverse Events

A formal quantitative network meta‐analysis could not be performed for safety due to the substantial heterogeneity in reporting formats (ranging from qualitative descriptions to inconsistent incidence rates) across trials. Instead, descriptive analysis was performed. Importantly, no cases of life‐threatening or serious adverse events (SAEs) were documented in any of the included RCTs. Regarding topical interventions, localized adverse reactions were the most frequently reported, including scalp irritation, pruritus, and transient erythema, particularly in individuals receiving microneedling and minoxidil. Systemic effects were primarily associated with oral pharmacotherapies. Oral minoxidil exhibited dose‐dependent hypertrichosis (e.g., 27% in the 1 mg group vs. 4% in the 0.25 mg group), while spironolactone combinations were linked to menstrual irregularities in approximately 13%–40% of participants. Notably, these safety findings are limited by potential under‐reporting and short follow‐up durations across the original studies. The documented adverse events are summarized in Table S13.

## 4 Discussion

This NMA integrated direct and indirect evidence from randomized trials to rank 15 interventions for FPHL. The majority of the trials included in this study adhered to a 24‐week protocol, thereby ensuring that comparisons were made at a consistent physiological stage of the hair cycle. However, given that the analysis excluded placebo comparators, all findings should be interpreted as relative efficacy rankings rather than absolute clinical benefits. The evidence certainty was rated as low to very low due to risk of bias and sparse data. Therefore, these SUCRA rankings should be interpreted as exploratory and hypothesis‐generating findings.

The combination of physical modalities with minoxidil appeared to provide significant benefits. MN + MX ranked highest for hair density (SUCRA = 0.93), while PLLA+MX ranked first for hair diameter (SUCRA = 0.97). The favorable rankings of MN + MX and PLLA+MX are biologically plausible, potentially reflecting synergistic mechanisms. It has been demonstrated that both MN and PLLA result in controlled micro‐injuries within the scalp. This has been demonstrated to enhance the penetration and absorption of topical minoxidil, while also stimulating localized wound healing responses [31, 32]. The aforementioned responses are characterized by an augmentation in perifollicular blood flow, a release of growth factors, and an activation of follicular stem cells [33]. Collectively, these phenomena promote hair growth and quality. It is noteworthy that PLLA+MX demonstrated higher efficacy than MN + MX in enhancing hair diameter [34], which may be attributable to its prolonged bioactive stimulation in comparison to the transient micro‐injuries induced by MN. These treatments offer a multifaceted therapeutic strategy for managing both hair loss and diameter reduction. The network's uncertainty should be considered when interpreting these findings. SUCRA rankings indicated probable relative efficacy of MN + MX and PLLA+MX, but these rankings must be considered with overlapping credible intervals. In sparse networks, SUCRA values can be affected by small‐study effects, and a high ranking doesn't guarantee a statistically significant clinical difference. The ranking is a hypothesis‐generating finding to indicate effective treatments, not a definitive conclusion.

Although prior systematic reviews by Adil et al. [10], Gupta et al. [11, 12], and Zhou et al. [13] have established efficacy profiles for non‐transplant androgenetic alopecia therapies, our study extends the evidence base through three key innovations. Regarding the study population, this research established a dedicated evidence network for FPHL, overcoming conventional limitations of male‐dominant focus or mixed‐population analyses. Therapeutically, our study evaluated 15 treatment regimens, including eight physical‐drug combinations, surpassing the six interventions assessed by Gupta et al. [11] and providing preliminary SUCRA‐based rankings for novel combinations such as MN and PLLA. However, these rankings need to be validated in larger, methodologically rigorous trials before any clinical inference can be drawn. For dynamic efficacy profiling, our findings tentatively suggest that MN + MX may compensate for the attenuating effects of MX reported by Feldman et al. [35], though validation in extended‐duration trials remains warranted. In endpoint selection, moving beyond the conventional focus on hair count metrics, we incorporated patient satisfaction as a primary outcome, directly addressing quality‐of‐life priorities in FPHL management, albeit with low‐certainty evidence.

In order to justify the transitivity assumption, the baseline severity, treatment duration, and protocols were evaluated across studies. In order to address the clinical heterogeneity, the mean change from baseline was utilized in order to account for initial numerical imbalances, and a random‐effects model was applied. In addition, sensitivity analyses were performed for the methodologies employed in hair density and diameter assessment to validate the mean differences (MD). The consistency observed between primary results and the automated‐imaging subset corroborated the robustness of our findings, particularly for MN + MX, thereby addressing concerns related to technical heterogeneity.

This study has certain limitations. Due to the absence of placebo or no‐treatment arms, the findings only reflect relative rankings relative to the current standard of care rather than absolute success rates. In the absence of a baseline placebo, it remains challenging to differentiate genuine therapeutic effects from natural hair loss progression. Moreover, no quantitative safety meta‐analysis was performed for safety due to heterogeneous reporting. Although most adverse events were mild, treatment strategies should be carefully individualized for patients given the systemic effects related to spironolactone (e.g., menstrual disorder) and oral minoxidil (e.g., dose‐dependent hypertrichosis). Finally, due to the limited, non‐validated data on patient satisfaction, the rankings of interventions for patient satisfaction should be regarded as supportive findings rather than definitive conclusions for guiding clinical decision‐making.

## 5 Conclusion

This NMA provides preliminary, hypothesis‐generating relative rankings of treatments for FPHL. Critically, due to no placebo arms, the findings only reflect relative comparisons, rather than indicating absolute clinical benefits. While MN + MX and PLLA+MX ranked highest for treatment efficacy and patient satisfaction, the results remain highly uncertain due to sparse data and heterogeneity in baseline severity and measurement techniques. These rankings represent exploratory findings rather than definitive clinical superiority. In the future, placebo‐controlled, standardized trials are needed to establish robust, clinically applicable risk–benefit profiles of treatment strategies for FPHL.

## Author Contributions

Conceptualization: Caixia Hu; Methodology: Caixia Hu; Formal analysis and investigation: Xin Li; Writing – original draft preparation: Xin Li, Lijing Lv, Xiaomei Han; Writing – review and editing: Yang Gao, Molin Yang; Funding acquisition: Yi Cheng; Resources: Caixia Hu; Supervision: Yi Cheng, Wenqing Wang and all authors commented on previous versions of the manuscript. All authors read and approved the final manuscript.

## Funding

This work was supported by the Hebei Natural Science Foundation (H2021206253).

## Ethics Statement

The authors have nothing to report.

## Conflicts of Interest

The authors declare no conflicts of interest.

## Supporting information


**Figure S1:** Cumulative ranking (SUCRA) curves for change in hair density. Interventions are labeled as follows: A, MX; B, 0.25 mg OM; C, 1 mg OM; D, MTF; E, C + MX; F, cow placenta; G, LLLT; H, LLLT+MX; I, LLLT+PRP; J, MN + MX; K, MN + YF; L, F + MX; M, PLLA+MX; N, PRP; O, SPT + MX. Full intervention names and abbreviations are provided in Figure 3.
**Figure S2:** Cumulative ranking (SUCRA) curves for change in hair diameter. Interventions are labeled as follows: A, MX; B, 0.25 mg OM; G, LLLT; H, LLLT+MX; I, LLLT+PRP; J, MN + MX; K, MN + YF; L, F 0.25% + MX; M, PLLA+MX; N, PRP. Full intervention names and abbreviations are provided in Figure 3.
**Figure S3:** Cumulative ranking (SUCRA) curves for patient satisfaction. Interventions are labeled as follows: A, MX; G, LLLT; H, LLLT+MX; J, MN + MX; K, MN + YF; M, PLLA+MX. Full intervention names and abbreviations are provided in Figure 3.
**Table S1:** Search strategy.
**Table S2:** Summary of baseline severity, treatment duration, and intervention protocols for transitivity assumption.
**Table S3:** League table for change in hair density.
**Table S4:** SUCRA values.
**Table S5:** League table for change in hair diameter.
**Table S6:** League table for patient satisfaction.
**Table S7:** Sensitivity analysis by method: direct comparisons for hair density (intervention vs. MX).
**Table S8:** Sensitivity analysis by method: direct comparisons for hair diameter (intervention vs. MX).
**Table S9:** Sensitivity analysis by study quality: direct comparisons for hair density (intervention vs. MX).
**Table S10:** Sensitivity analysis by study quality: direct comparisons for hair diameter (intervention vs. MX).
**Table S11:** Sensitivity analysis by study quality: direct comparisons for patient satisfaction (intervention vs. MX).
**Table S12:** GRADE assessment of evidence certainty for key comparisons.
**Table S13:** Summary of reported adverse events across included studies.

## Data Availability

The data that support the findings of this study are available from the corresponding author (Dr. Yi Cheng) upon reasonable request.
