# Supplementary material for: Comparative Efficacy of Non‐Transplant Interventions for Female Pattern Hair Loss: A Systematic Review and Network Meta‐Analysis
Source: J Cosmet Dermatol. 2026 Jul 12;25(7):e71037. doi: 10.1111/jocd.71037 (PMC13357034; doi:10.1111/jocd.71037)
Supplement: Supplementary file 1 — Figure S1: Cumulative ranking (SUCRA) curves for change in hair density. Interventions are labeled as follows: A, MX; B, 0.25 mg OM; C, 1 mg OM; D, MTF; E, C + MX; F, cow placenta; G, LLLT; H, LLLT+MX; I, LLLT+PRP; J, MN + MX; K, MN + YF; L, F + MX; M, PLLA+MX; N, PRP; O, SPT + MX. Full intervention names and abbreviations are provided in Figure 3. Figure S2: Cumulative ranking (SUCRA) curves for change in hair diameter. Interventions are labeled as follows: A, MX; B, 0.25 mg OM; G, LLLT; H, LLLT+MX; I, LLLT+PRP; J, MN + MX; K, MN + YF; L, F 0.25% + MX; M, PLLA+MX; N, PRP. Full intervention names and abbreviations are provided in Figure 3. Figure S3: Cumulative ranking (SUCRA) curves for patient satisfaction. Interventions are labeled as follows: A, MX; G, LLLT; H, LLLT+MX; J, MN + MX; K, MN + YF; M, PLLA+MX. Full intervention names and abbreviations are provided in Figure 3. Table S1: Search strategy. Table S2: Summary of baseline severity, treatment duration, and intervention protocols for transitivity assumption. Table S3: League table for change in hair density. Table S4: SUCRA values. Table S5: League table for change in hair diameter. Table S6: League table for patient satisfaction. Table S7: Sensitivity analysis by method: direct comparisons for hair density (intervention vs. MX). Table S8: Sensitivity analysis by method: direct comparisons for hair diameter (intervention vs. MX). Table S9: Sensitivity analysis by study quality: direct comparisons for hair density (intervention vs. MX). Table S10: Sensitivity analysis by study quality: direct comparisons for hair diameter (intervention vs. MX). Table S11: Sensitivity analysis by study quality: direct comparisons for patient satisfaction (intervention vs. MX). Table S12: GRADE assessment of evidence certainty for key comparisons. Table S13: Summary of reported adverse events across included studies. [file JOCD-25-e71037-s001.docx]

**Supplementary Table S1** Search strategy

(((randomized controlled trial[Publication Type] OR randomized[Title/Abstract] OR

placebo[Title/Abstract])) AND ((((female[Title/Abstract]) OR (females[Title/Abstract]))

OR ("Female"[Mesh])) AND (((((((((((((((((((((((((Baldness[Title/Abstract]) OR (Hair

Loss[Title/Abstract])) OR (Hair Losses[Title/Abstract])) OR (Loss, Hair[Title/Abstract])) OR (Losses, Hair[Title/Abstract])) OR (Alopecia, Male Pattern[Title/Abstract])) OR (Male Pattern Alopecia[Title/Abstract])) OR (Baldness, Male Pattern[Title/Abstract])) OR (Male Pattern Baldness[Title/Abstract])) OR (Female Pattern Baldness[Title/Abstract])) OR

(Baldness, Female Pattern[Title/Abstract])) OR (Androgenetic Alopecia[Title/Abstract]))

OR (Pattern Baldness[Title/Abstract])) OR (Androgenic Alopecia[Title/Abstract])) OR

(Baldness, Pattern[Title/Abstract])) OR (Alopecia, Androgenic[Title/Abstract])) OR

(Alopecias, Androgenic[Title/Abstract])) OR (Androgenic Alopecias[Title/Abstract])) OR

(Alopecia, Androgenetic[Title/Abstract])) OR (Pseudopelade[Title/Abstract])) OR

(Alopecia Cicatrisata[Title/Abstract])) OR (Alopecia Cicatrisatas[Title/Abstract])) OR ("Alopecia"[Mesh])))))

**Supplementary Table S2.** Summary of baseline severity, treatment duration, and intervention protocols for transitivity evaluation.

| Study (ID) | Mean Age (Years) | Baseline Severity (Scale) | Baseline Hair Density (hairs/cm²) | Duration (Weeks) | Intervention & Protocol Details (Dosage/Frequency) |
| --- | --- | --- | --- | --- | --- |
| Zhang 2022 | 30.0–31.6 | Sinclair II–IV | 83.80 ± 12.83 to 84.50 ± 15.20 | 24 | 2% minoxidil (1ml) vs. 2% minoxidil + Microneedle (1ml + twice/week) |
| Nascimento 2022 | 41.4–41.7 | Sinclair II–V | 163.2 ± 46.0 to 164.6 ± 48.1 | 24 | Oral Minoxidil (0.25mg tablet/day) vs. Oral Minoxidil (1mg tablet/day) |
| Esmat 2017 | 31.2–35.6 | Ludwig I–III | 141.93 ± 19.43 to 145.47 ± 20.37 | 24 | 5% minoxidil (1ml) vs. LLLT (once/day) vs. LLLT + 5% minoxidil (1ml + once/day) |
| Bassiouny 2022 | 36.7–38.6 | Sinclair II–IV | 97.62 ± 32.89 to 107.87 ± 28.63 | 24 | 5% minoxidil + 1% cetirizine (1ml) vs. 5% minoxidil (1ml) (Forehead & Vertex groups) |
| Yang Liu 2020 | 20.0–49.0 | Ludwig I–III | 75.90 ± 7.55 to 80.50 ± 6.75 | 24 | LLLT (once/day) vs. 5% minoxidil (1ml) vs. LLLT + 5% minoxidil (once/day + 1ml) |
| Xuelei Liang 2022 | 30.8–31.6 | Sinclair II–III | 99.14 ± 15.85 to 101.37 ± 17.32 | 24 | 5% minoxidil (1ml) vs. 5% minoxidil + Microneedle (1ml, twice/week) vs. Spironolactone (100mg) + Microneedle (1ml + twice/week) |
| Liang 2022 | 31.0–31.6 | Sinclair II–III | 99.18 ± 19.38 to 101.37 ± 17.32 | 24 | 5% minoxidil (1ml) vs. YF + Microneedle (1ml, twice/week) vs. 5% minoxidil + Microneedle (1ml, twice/week) |
| Khattab 2019 | 21.0–49.0 | Ludwig I–III | 113.00 ± 24.00 to 114.00 ± 27.00 | 24 | Monofilament therapy + 2% minoxidil (1ml) vs. 2% minoxidil (1ml) |
| Ramos 2020 | 40.6–47.3 | Sinclair I–III | 163.2 ± 46.0 to 164.6 ± 48.1 | 24 | Oral Minoxidil (1mg tablet/day) vs. 5% topical minoxidil (once/day) |
| Suchonwanit 2018 | 56.8–59.8 | Ludwig I–III | 98.1 ± 19.0 to 102.5 ± 22.7 | 24 | 0.25% finasteride + 3% minoxidil (1ml) vs. 3% minoxidil (1ml) |
| Blume 2011 | 23.0–75.0 | Savin III–VI | 164.0 ± 43.0 to 176.2 ± 44.9 | 24 | 5% minoxidil (50mg) vs. 2% minoxidil (1ml) |
| Vahabi 2021 | 18.0–50.0 | Sinclair II–V | 100.8 ± 154.8 to 102.0 ± 50.7 | 36 | Oral Minoxidil (0.25mg tablet/day) vs. 2% topical minoxidil (1ml) |
| Barat 2020 | 41.2–44.3 | Sinclair II–V | Not Reported | 24 | 2% minoxidil (1ml) vs. Bovine placenta serum extract |

**Supplementary Table S3.** League table of pairwise comparisons for change in hair density

| MD 95%CrI | | | | | | | | | | | | | | |
| --- | --- | --- | --- | --- | --- | --- | --- | --- | --- | --- | --- | --- | --- | --- |
| MX |  |  |  |  |  |  |  |  |  |  |  |  |  |  |
| -3.68(-43.29, 34.56) | 0.25mgOM |  |  |  |  |  |  |  |  |  |  |  |  |  |
| 9.29(-17.60, 37.74) | 13.69(-21.42, 51.24) | 1mgOM |  |  |  |  |  |  |  |  |  |  |  |  |
| 3.46(-7.13, 13.95) | 7.23(-32.40, 47.31) | -5.89(-36.05, 23.27) | 5%MTF |  |  |  |  |  |  |  |  |  |  |  |
| -1.26(-16.95, 14.65) | 2.83(-39.30, 44.88) | -10.74(-43.81, 20.48) | -4.64(-23.59, 14.42) | 1%C+MX |  |  |  |  |  |  |  |  |  |  |
| -0.70(-9.07, 7.76) | 3.14(-35.80, 42.98) | -10.03(-39.63, 18.30) | -4.16(-17.44, 9.25) | 0.55(-17.24, 18.07) | Cow-placenta |  |  |  |  |  |  |  |  |  |
| 0.84(-6.36, 7.95) | 4.59(-34.24, 44.47) | -8.43(-37.69, 19.24) | -2.63(-15.36, 10.16) | 2.19(-15.14, 19.04) | 1.54(-9.56, 12.43) | LLLT |  |  |  |  |  |  |  |  |
| 12.80(5.65, 20.02) | 16.62(-22.55, 56.57) | 3.55(-25.83, 31.26) | 9.37(-3.28, 22.13) | 14.16(-3.15, 31.13) | 13.51(2.47, 24.53) | 11.97(5.00, 19.10) | LLLT+MX |  |  |  |  |  |  |  |
| 6.28(-4.70, 17.10) | 10.09(-29.78, 50.64) | -2.97(-33.28, 25.61) | 2.84(-12.21, 17.82) | 7.61(-11.46, 26.08) | 6.96(-6.92, 20.59) | 5.43(-2.84, 13.58) | -6.55(-17.51, 4.16) | LLLT+PRP |  |  |  |  |  |  |
| 21.13(15.38, 27.30) | 25.00(-13.49, 64.60) | 11.95(-17.25, 39.24) | 17.71(5.90, 30.04) | 22.50(5.63, 39.11) | 21.83(11.97, 32.37) | 20.30(11.40, 29.94) | 8.33(-0.68, 17.82) | 14.87(2.93, 27.66) | MN+MX |  |  |  |  |  |
| -3.61(-11.77, 4.87) | 0.21(-38.92, 39.93) | -12.89(-42.71, 15.15) | -7.08(-20.29, 6.51) | -2.28(-20.06, 15.12) | -2.92(-14.53, 8.98) | -4.46(-15.18, 6.66) | -16.45(-27.26, 5.36) | -9.89(-23.35, 4.07) | -24.75(-33.42, 16.36) | MN+YF |  |  |  |  |
| 3.67(-33.90, 39.10) | 7.65(-46.61, 62.03) | -6.09(-54.069, 38.88) | 0.22(-38.58, 37.00) | 5.06(-35.93, 43.27) | 4.38(-34.29, 40.51) | 2.81(-35.43, 38.79) | -9.20(-47.31, 26.91) | -2.59(-42.02, 34.34) | -17.50(-55.64, 18.30) | 7.23(-31.26, 43.37) | 0.25%F+MX |  |  |  |
| 18.88(4.03, 33.70) | 22.84(-18.95, 64.35) | 9.58(-22.61, 40.87) | 15.34(-2.64, 33.62) | 20.14(-1.86, 41.89) | 19.53(2.63, 36.37) | 18.05(1.76, 34.50) | 6.06(-10.32, 22.43) | 12.59(-5.35, 30.74) | -2.29(-18.65, 13.42) | 22.52(5.38, 39.30) | 15.06(-23.57, 55.60) | PLLA+MX |  |  |
| -0.96(-11.90, 9.75) | 2.75(-36.84, 43.44) | -10.24(-40.59, 18.40) | -4.43(-19.59, 10.57) | 0.38(-18.65, 18.79) | -0.28(-14.07, 13.33) | -1.82(-10.04, 6.35) | -13.78(-24.70, 3.13) | -7.24(-15.46, 0.98) | -22.12(-34.80, 10.17) | 2.65(-11.30, 16.10) | -4.63(-41.56, 34.67) | -19.82(-37.91, 1.93) | PRP |  |
| 7.08(-1.16, 15.81) | 10.80(-28.18, 50.68) | -2.24(-31.89, 26.17) | 3.59(-9.70, 17.28) | 8.40(-9.45, 25.95) | 7.75(-3.95, 19.84) | 6.23(-4.61, 17.57) | -5.74(-16.57, 5.55) | 0.79(-12.53, 14.87) | -14.05(-22.85, 5.44) | 10.70(-0.46, 22.04) | 3.58(-32.96, 42.12) | -11.75(-28.80, 5.57) | 8.03(-5.32, 22.17) | SPT+MX |

**Supplementary Table S4** SUCRA values

| Intervetion | MX | 0.25mgOM | 1mgOM | 5%MTF | 1%C+MX | Cow-placenta | LLLT | LLLT+MX | LLLT+PRP | MN+MX | MN+YF | 0.25%F+MX | PLLA+MX | PRP | SPT+MX |
| --- | --- | --- | --- | --- | --- | --- | --- | --- | --- | --- | --- | --- | --- | --- | --- |
| Changes in Hair Density | 0.33 | 0.34 | 0.62 | 0.48 | 0.32 | 0.30 | 0.38 | 0.79 | 0.60 | 0.93 | 0.18 | 0.44 | 0.88 | 0.27 | 0.63 |
| Hair diameter changes | 0.24 | 0.31 | NA | NA | NA | NA | 0.55 | 0.67 | 0.66 | 0.32 | 0.20 | 0.45 | 0.97 | 0.64 | NA |
| Patient satisfaction | 0.17 | NA | NA | NA | NA | NA | 0.14 | 0.57 | NA | 0.98 | 0.55 | NA | 0.58 | NA | NA |

**Supplementary Table S5** League table of pairwise comparisons for change in hair diameter

| MD 95%CrI | | | | | | | | | |
| --- | --- | --- | --- | --- | --- | --- | --- | --- | --- |
| MX |  |  |  |  |  |  |  |  |  |
| 1.01(-27.46, 28.97) | 0.25mgOM |  |  |  |  |  |  |  |  |
| 7.80(-10.87, 30.32) | 6.67(-25.93, 43.65) | LLLT |  |  |  |  |  |  |  |
| 10.79(-9.40, 31.75) | 9.77(-24.85, 45.24) | 2.96(-19.33, 22.14) | LLLT+MX |  |  |  |  |  |  |
| 10.46(-21.86, 47.52) | 9.44(-32.75, 57.09) | 2.84(-25.06, 31.06) | -0.25(-33.31, 36.74) | LLLT+PRP |  |  |  |  |  |
| 1.01(-27.35, 29.06) | -0.01(-39.71, 40.14) | -6.82(-43.85, 26.03) | -9.81(-44.99, 24.67) | -9.57(-56.93, 32.89) | MN+MX |  |  |  |  |
| -1.94(-29.97, 26.00) | -2.96(-42.66, 36.89) | -9.74(-46.66, 23.03) | -12.76(-48.39, 21.67) | -12.49(-60.08, 30.17) | -2.97(-31.36, 25.16) | MN+YF |  |  |  |
| 4.91(-23.29, 33.09) | 3.91(-36.11, 44.27) | -2.85(-39.87, 30.05) | -5.90(-41.12, 28.74) | -5.6(-52.83, 36.48) | 3.87(-35.81, 43.74) | 6.87(-32.83, 46.55) | 0.25%F+MX |  |  |
| 43.63(15.35, 71.80) | 42.64(2.53, 82.38) | 35.85(-1.22, 68.70) | 32.83(-2.74, 67.22) | 33.09(-14.49, 75.51) | 42.62(2.42, 82.25) | 45.64(5.34, 85.08) | 38.72(-1.65, 78.63) | PLLA+MX |  |
| 10.03(-22.16, 47.21) | 8.97(-33.00, 56.05) | 2.35(-25.28, 30.61) | -0.71(-33.71, 36.20) | -0.48(-28.53, 27.77) | 9.08(-33.12, 56.30) | 12.01(-30.35, 59.33) | 5.14(-36.94, 52.48) | -33.58(-75.51, 13.87) | PRP |

**Supplementary Table S6** League table of pairwise comparisons for patient satisfaction

| **Log (OR)** 95% CrI | | | | | |
| --- | --- | --- | --- | --- | --- |
| MX |  |  |  |  |  |
| . (.,.) | LLLT |  |  |  |  |
| 0.29(-3.17, 3.75) | 2.16(-0.89, 5.22) | LLLT+MX |  |  |  |
| 2.96(0.06, 5.86) | 2.96(-0.39, 6.31) | 0.80(-3.41, 5.01) | MN+MX |  |  |
| -1.78(-3.60, 0.03) | 1.78(-0.69, 4.26) | -0.38(-3.93, 3.18) | -1.18(-4.41, 2.06) | MN+YF |  |
| 2.45(0.83, 4.08) | 2.45(0.12, 4.79) | 0.29(-3.17, 3.75) | -0.51(-3.83, 2.82) | 0.67(-1.77, 3.11) | PLLA+MX |

**Supplementary Table S7.** Sensitivity analysis by method: direct comparisons for hair density (intervention vs MX)

| **Study** | **Intervention** | **Mean Difference (hairs/cm²)** | **95% CI** | **Significance** |
| --- | --- | --- | --- | --- |
| Blume‑Peytavi 2011 | D (5% minoxidil foam) | 3.5 | -4.0 – 11.0 | NS |
| Iamsumang 2018 | L (0.25% finasteride + MX) | 2.8 | -33.22 – 38.82 | NS |
| Ramos 2020 | C (1 mg oral minoxidil) | 7.0 | -22.52 – 36.52 | NS |
| Xuelei Liang 2022 | O (spironolactone + MX) | 6.81 | 1.83 – 11.79 | S |
| Xuelei Liang 2022 | **J (microneedling + MX)** | **20.38** | **14.67 – 26.09** | **S** |
| Yao Zhang 2022 | **J (microneedling + MX)** | **24.85** | **14.76 – 34.94** | **S** |
| Sadegh Vahabi 2021 | B (0.25 mg oral minoxidil) | 7.6 | -50.25 – 65.45 | NS |
| Esmat 2017 | G (low‑level laser therapy) | 0.9 | -12.37 – 14.17 | NS |
| Esmat 2017 | H (LLLT + MX) | 13.4 | 0.24 – 26.56 | S |

*S = significant (95% CI does not include 0); NS = non-significant.

**Supplementary Table S8** Sensitivity analysis by method: direct comparisons for hair diameter (intervention vs MX)

| **Study** | **Intervention** | **Mean Difference (µm)** | **95% CI** | **Significance** |
| --- | --- | --- | --- | --- |
| Iamsumang 2018 | L (0.25% finasteride + MX) | 4.9 | 1.01 – 8.79 | S |
| Sadegh Vahabi 2021 | B (0.25 mg oral minoxidil) | 1.0 | -0.95 – 2.95 | NS |
| Esmat 2017 | G (low‑level laser therapy) | 14.0 | 1.05 – 26.95 | S |
| Esmat 2017 | H (LLLT + MX) | 12.0 | -3.09 – 27.09 | NS |

Note: PLLA+MX (M) could not be assessed because the only study (Khattab 2019) was excluded due to lack of automated trichoscopy and fixed target site.

**Supplementary Table S9** Sensitivity analysis by study quality: direct comparisons for hair density (intervention vs MX)

| **Study** | **Intervention** | **Mean Difference (hairs/cm²)** | **95% CI** | **Significance** |
| --- | --- | --- | --- | --- |
| Blume‑Peytavi 2011 | D (5% minoxidil foam) | 3.5 | -4.0 – 11.0 | NS |
| Iamsumang 2018 | L (0.25% finasteride + MX) | 2.8 | -33.22 – 38.82 | NS |
| Ramos 2020 | C (1 mg oral minoxidil) | 7.0 | -22.52 – 36.52 | NS |
| Xuelei Liang 2022 | O (spironolactone + MX) | 6.81 | 1.83 – 11.79 | S |
| Xuelei Liang 2022 | **J (microneedling + MX)** | **20.38** | **14.67 – 26.09** | **S** |
| Yao Zhang 2022 | **J (microneedling + MX)** | **24.85** | **14.76 – 34.94** | **S** |
| Sadegh Vahabi 2021 | B (0.25 mg oral minoxidil) | 7.6 | -50.25 – 65.45 | NS |
| Esmat 2017 | G (low‑level laser therapy) | 0.9 | -12.37 – 14.17 | NS |
| Esmat 2017 | H (LLLT + MX) | 13.4 | 0.24 – 26.56 | S |

*S = significant (95% CI does not include 0); NS = non-significant.

**Supplementary Table S10** Sensitivity analysis by study quality: direct comparisons for hair diameter (intervention vs MX)

| **Study** | **Intervention** | **Mean Difference (µm)** | **95% CI** | **Significance** |
| --- | --- | --- | --- | --- |
| Iamsumang 2018 | L (0.25% finasteride + MX) | 4.9 | 1.01 – 8.79 | S |
| Sadegh Vahabi 2021 | B (0.25 mg oral minoxidil) | 1.0 | -0.95 – 2.95 | NS |
| Esmat 2017 | G (low‑level laser therapy) | 14.0 | 1.05 – 26.95 | S |
| Esmat 2017 | H (LLLT + MX) | 12.0 | -3.09 – 27.09 | NS |

Note: PLLA+MX (M) was excluded because the only study (Khattab 2019) was rated at high risk of bias (split scalp design).

**Supplementary Table S11** Sensitivity analysis by study quality: direct comparisons for patient satisfaction (intervention vs MX)

| **Study** | **Comparison** | **Risk Ratio (RR)** | **95% CI** | **Significance** |
| --- | --- | --- | --- | --- |
| Zhuo 2022 | **J (microneedling + MX) vs A** | **1.23** | **1.06 – 1.42** | **S** |
| Zhuo 2022 | K (microneedling + Yu Fa) vs A | 1.13 | 0.97 – 1.32 | NS |
| Esmat 2017 | G (LLLT) vs A | 1.00 | 0.68 – 1.47 | NS |
| Esmat 2017 | **H (LLLT + MX) vs A** | **1.25** | **1.00 – 1.56** | **S** (borderline) |

Note: RR >1 favours intervention; S = significant (95% CI does not include 1); NS = non-significant.

**Supplementary Table S12**. GRADE assessment of evidence certainty for key comparisons

| Comparison | Outcome | No. of studies | Total participants | Effect estimate (MD) | 95% CI | Certainty of evidence | Main reasons for downgrading |
| --- | --- | --- | --- | --- | --- | --- | --- |
| MN+MX vs MX | Hair density | 5 | ~320 | MD = 21.13 | 15.38 to 27.30 | Low | Risk of bias (lack of blinding), Imprecision |
| PLLA+MX vs MX | Hair density | 1 | ~60 | MD = 18.88 | 4.03 to 33.70 | Very low | Imprecision, Risk of bias, Suspected publication bias |
| PLLA+MX vs MX | Hair diameter | 1–2 | <100 | MD = 43.63 | 15.35 to 71.80 | Very low | Imprecision, Risk of bias, Indirectness |
| MN+MX vs MX | Patient satisfaction | 2 | ~150 | MD = 2.96 | 0.06 to 5.86 | Very low | Imprecision, Indirectness (non-validated scale), Detection bias |
| MN+MX vs LLLT+MX | Hair density | 1 | ~80 | MD = 8.33 | 0.68 to 17.82 | Very low | Single study, Imprecision, High risk of bias |

**Supplementary Table S13** Summary of reported adverse events across included studies

| **Study** | **Intervention Groups** | **Reported Adverse Events (n, %)** | **Key Safety Observations** |
| --- | --- | --- | --- |
| Liang 2022 | MN+YF vs. MN+MX vs. MX | MN+YF: 10.5%; MN+MX: 60.0%; MX: 71.1%. | Botanical (YF) significantly reduced dandruff vs. MX groups (P<0.05). |
| Xuelei Liang 2022 | MX+SPT vs. MX+MN vs. MX | MX+SPT: Menstrual disorder (13%), Hyperkalemia, Edema. MX+MN: Pruritus, Infection (n=1). MX:Pruritus. | Systemic effects (menstrual/electrolyte) were exclusive to the SPT group. |
| Yang Liu 2020 | LLLT+MX vs. MX vs. LLLT | Combined: Tenderness (7.7%), Pruritus (11.5%). MX:Pruritus (13.8%). LLLT: Tenderness (3.6%). | Localized symptoms were mild and resolved within 2 weeks. |
| Nascimento 2022 | Oral MX 0.25mg vs. Oral MX 1mg | 1mg: Hypertrichosis (27%), Edema (4%), HR increase (6.5%). 0.25mg: Hypertrichosis (4%). | Dose-dependent increase in hypertrichosis (P=0.02) and heart rate (P<0.01). |
| Yao Zhang 2022 | MN+MX vs. MX | MN+MX: Hypertrichosis (25%), Headache (5%), Erythema. MX: Hypertrichosis (7.5%), Pruritus (17.5%). | MN erythema was transient; headache resolved within 24 hours. |
| Vahabi 2021 | Oral MX 0.25mg vs. MX | Oral: Hirsutism (7.7%), GI intolerance (7.7%), Hypotension (3.8%). Topical: Hirsutism (8.0%). | Two patients in the oral group withdrew due to GI intolerance. |
| Ramos 2020 | Oral MX 1mg vs. MX | Oral: Hypertrichosis (27%), Edema (4%), HR increase (6.5%). Topical: Pruritus (19%), Hypertrichosis (4%). | No SAEs; hypertrichosis was mild and clinically manageable. |
| Esmat 2017 | LLLT+MX vs. MX vs. LLLT | Combined: Shedding (60%), Irritation (27%), Tenderness (40%). MX: Shedding (80%), Irritation (40%). LLLT: Tenderness (27%). | Initial shedding was prevalent in all MX-containing arms. |
| Bassiouny 2022 | MX+Cetirizine vs. MX | Both groups: Itching, dry hair, shedding, and dandruff. | No significant difference between groups in AE frequency. |
| Yang Liu 2020 (PRP) | LLLT+PRP vs. PRP vs. LLLT | Combined: Pain/swelling (10.5%), Tenderness (5.3%). PRP: Pain/swelling (16.7%). LLLT: Tenderness (5.3%). | Injection pain was transient and minimized by cooling/lidocaine. |
| Barat 2020 | Cow Placenta vs. MX 2% | MX: Pruritus/Irritation (19.4%), Hypertrichosis (9.7%). Placenta: Pruritus (2.3%). | Minimal AEs; none led to treatment discontinuation. |
| Suchonwanit 2018 | FMX+MX vs. MX | FMX+MX: Pruritus (n=2). MX: Pruritus (n=2), Irritation (n=1). | Serum DHT significantly decreased in FMX group (P=0.016). |
| Blume-Peytavi 2011 | 5% MX Foam vs. 2% MX Solution | 5%: Pruritus (16.1%), Shedding (12.5%). 2%: Pruritus (36.8%), Shedding (17.5%), Dandruff (17.5%). | 5% Foam showed significantly better local tolerance (P=0.046). |
| Khattab 2019 | PLLA+MX vs. MX | Combined: Pain (n=22), Erythema (n=18), Edema (n=7), Pruritus (n=8). | All discomfort resolved within 48-72 hours post-treatment. |

**
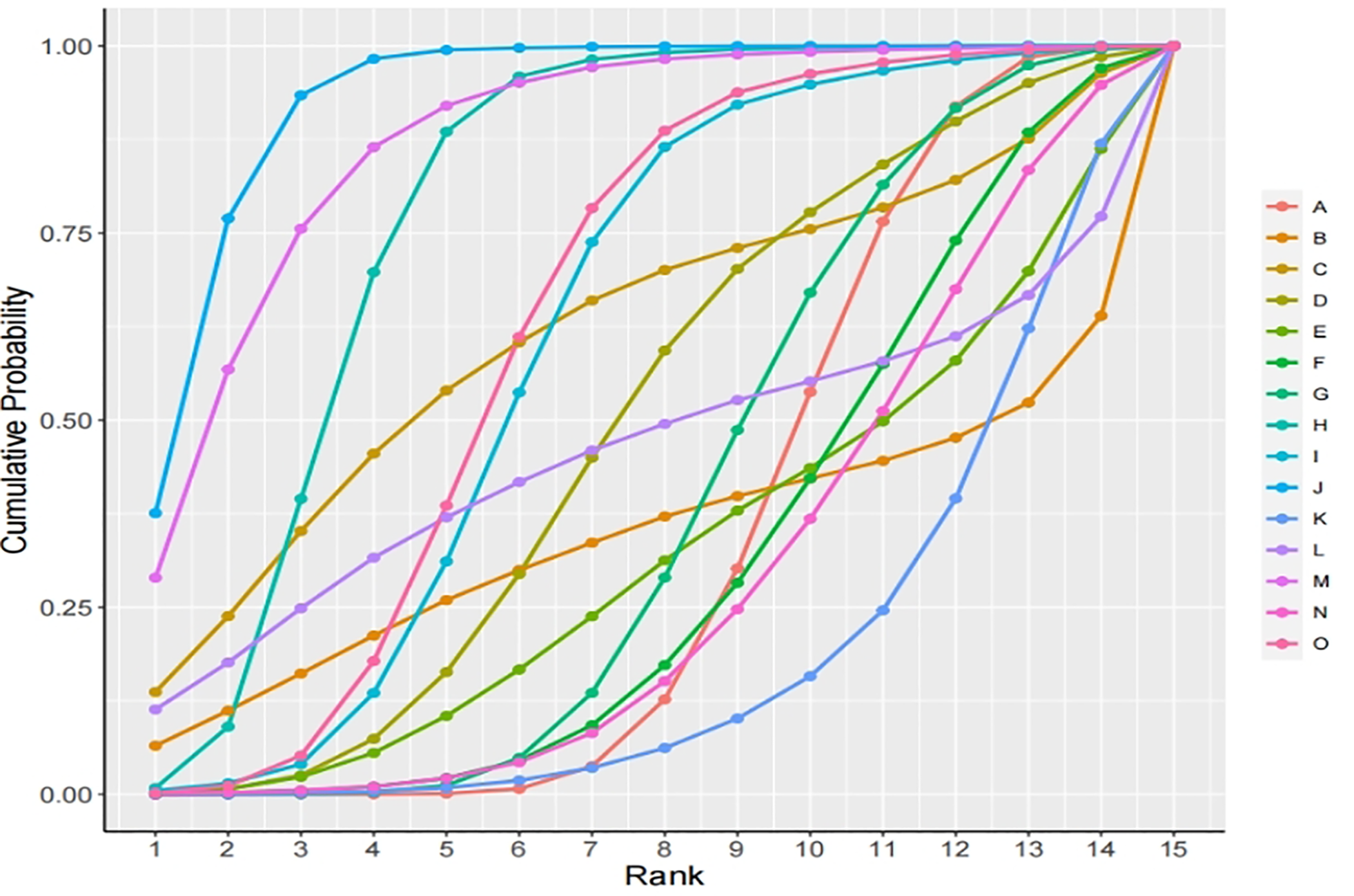
**

**Supplementary Figure S1.** Cumulative ranking (SUCRA) curves for change in hair density. Interventions are labeled as follows: A, MX; B, 0.25mg OM; C, 1mg OM; D, MTF; E, C+MX; F, cow placenta; G, LLLT; H, LLLT+MX; I, LLLT+PRP; J, MN+MX; K, MN+YF; L, F+MX; M, PLLA+MX; N, PRP; O, SPT+MX. Full intervention names and abbreviations are provided in Figure 3.

**
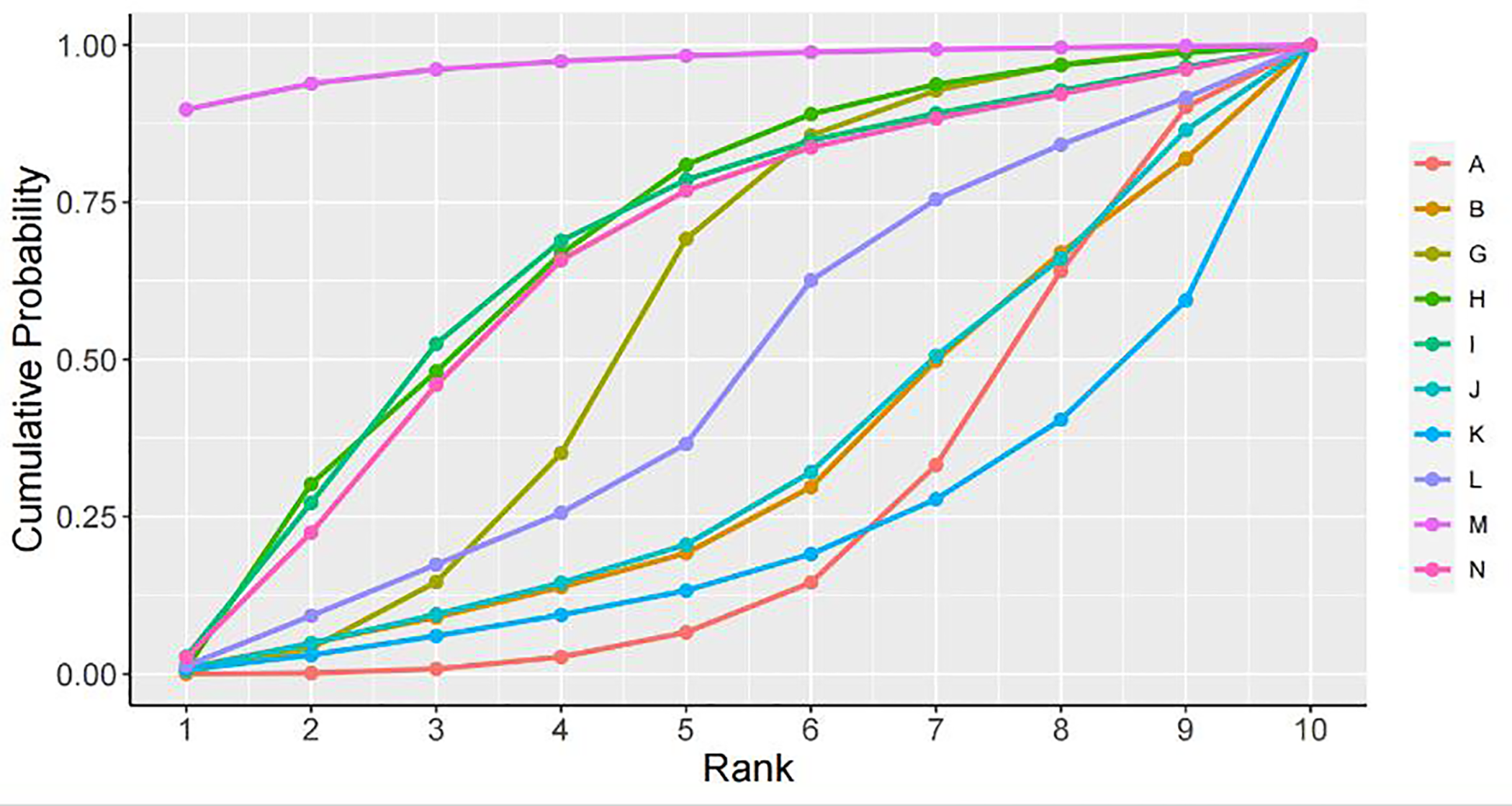
**

**Supplementary Figure S2.** Cumulative ranking (SUCRA) curves for change in hair diameter. Interventions are labeled as follows: A, MX; B, 0.25mg OM; G, LLLT; H, LLLT+MX; I, LLLT+PRP; J, MN+MX; K, MN+YF; L, F 0.25%+MX; M, PLLA+MX; N, PRP. Full intervention names and abbreviations are provided in Figure 3.

**
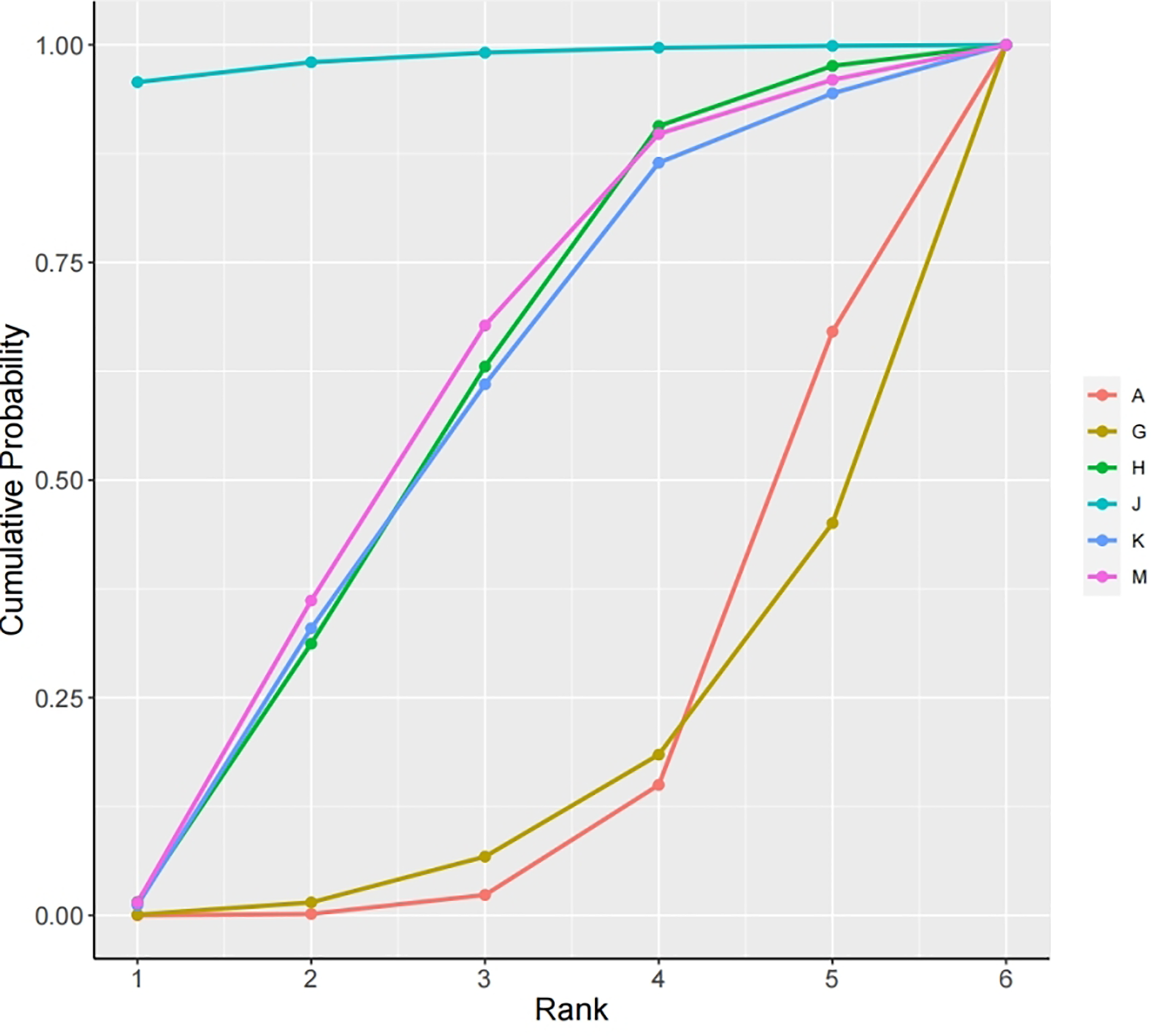
**

**Supplementary Figure S3**. Cumulative ranking (SUCRA) curves for patient satisfaction. Interventions are labeled as follows: A, MX; G, LLLT; H, LLLT+MX; J, MN+MX; K, MN+YF; M, PLLA+MX. Full intervention names and abbreviations are provided in Figure 3.
